# Exosomes From Human Urine-Derived Stem Cells Encapsulated Into PLGA Nanoparticles for Therapy in Mice With Particulate Polyethylene-Induced Osteolysis

**DOI:** 10.3389/fmed.2021.781449

**Published:** 2021-12-06

**Authors:** Hui Li, Yihe Hu, Min Zeng, Junxiao Yang, Xiaolei Fan, Yinan Wang, Jie Xie

**Affiliations:** ^1^Department of Orthopedics, Xiangya Hospital, Central South University, Changsha, China; ^2^Hunan Engineering Research Center of Biomedical Metal and Ceramic Implants, Xiangya Hospital, Central South University, Changsha, China

**Keywords:** exosomes, urine-derived stem cells, PLGA microsphere, nanoparticles, UHMWPE, wear particle-induced osteolysis

## Abstract

**Background:** Periprosthetic osteolysis is the primary reason for arthroplasty failure after total joint replacement because of the generation of wear particles and subsequent bone erosion around the prosthesis, which leads to aseptic loosening. Periprosthetic osteolysis is often treated with revision surgery because of the lack of effective therapeutic agents. As key messengers of intercellular interactions, exosomes can be independently used as therapeutic agents to promote tissue repair and regeneration. In this study, we fabricated poly(lactic-co-glycolic acid) (PLGA) nanoparticles (NPs) that carry exosomes derived from human urine stem cells (USC-Exos) and explored their effects on polyethylene-induced osteolysis.

**Methods:** USCs were identified by multipotent differentiation and flow cytometry analyses. USC-Exos were isolated and identified by transmission electron microscopy (TEM), dynamic light scattering (DLS), and western blotting. PLGA microspheres containing USC-Exos were fabricated to synthesize NPs using the mechanical double-emulsion method. The obtained NPs were characterized in terms of stability, toxicity, exosome release, and cell uptake. Then, these NPs were implanted into the murine air pouch model, and their effects on polyethylene-induced osteolysis were evaluated by microcomputed tomography (micro-CT) and histological analyses.

**Results:** The average NP diameter was ~282 ± 0.4 nm, and the zeta potential was −2.02 ± 0.03 mV. After long-term storage at room temperature and 4°C, the NP solution was stable without significant coaggregation. *In vitro* release profiles indicated sustained release of exosomes for 12 days. *In vivo*, injection of NPs into the murine air pouch caused less osteolysis than that of USC-Exos, and NPs significantly reduced bone absorption, as indicated by histology and micro-CT scanning.

**Conclusion:** Our findings suggest that USC-Exo-based PLGA NPs can prevent particulate polyethylene-induced osteolysis and bone loss.

## Introduction

Total joint replacement can effectively treat joint diseases such as osteoarthritis, complex fractures, and osteonecrosis (1, 2). The total number of joint replacement surgeries performed every year worldwide is ~1.5 million, and the demand for this treatment is expected to increase (3). Despite the continuous innovation of artificial prosthesis manufacturing technology and a comprehensive understanding of biomechanics, a substantial decline in revision rates or second operations has not been achieved over the last decade (4). The main reason for arthroplasty failure after total joint replacement is aseptic loosening, representing ~75% of all cases and severely impacting patients and the healthcare system (5). The basic mechanism of periprosthetic osteolysis mainly starts with the production of wear debris that induces macrophages or T lymphocytes to release a series of inflammatory factors, such as TNF-α and IL-6, which subsequently induce RANKL secretion, osteoclast formation, and finally bone resorption (6, 7). Therefore, inflammation, bone resorption, and foreign-body granuloma formation around the bone-implant interface represent vital factors leading to osteolysis (8). Presently, no effective agents or drugs to inhibit or treat periprosthetic osteolysis have been approved, and aseptic loosening can be treated only by revision surgery. An issue concerning surgical revision that warrants consideration by surgeons is the reduction in bone stock due to osteolysis defects and restoration of the integrity of the proximal femur and acetabulum. Hence, alternative therapeutic strategies for aseptic loosening are required to ameliorate bone loss and promote bone regeneration.

Recently, exosomes derived from multivesicular bodies (MVBs) have attracted increased attention in the field of tissue repair and regeneration (9). Endosome-derived exosomes are 40–150-nm nanoparticles that play important roles in regulating intercellular interactions by delivering biomolecules (e.g., RNA and protein) to recipient cells, in which biomolecules modulate the expression of target genes or proteins and subsequently influence the function of recipient cells (9). Exosomes are key intermediators of cellular communication (9, 10) and are released by various cell types (11, 12). Additionally, USCs are considered significantly superior to other stem cells because USCs can be isolated from collected human urine samples using noninvasive, simple, safe and low-cost methods (11). These advantages make USCs a promising cell source from which to isolate exosomes. Recently, researchers determined that exosomes obtained from human USCs (USC-Exos) significantly promote bone formation and revascularization and inhibit osteoclastic bone resorption (13, 14). In addition to their excellent therapeutic effect on tissue repair and regeneration, exosomes are regarded as promising biomaterials in the field of drug delivery systems because of their superior aqueous stability, biocompatibility and long blood circulation *in vivo* without obvious degeneration (15). Therefore, exosomes could be used as sustained-release therapeutic agents to design various drug delivery systems for regenerative medicine.

Nanoparticulate delivery systems are increasingly being used because of their advantages in potentially overcoming many inherent obstacles associated with using free drugs or growth factors. Poly(lactic-co-glycolic acid) (PLGA) has been approved by the United States Food and Drug Administration and European Medicines Agency for drug release and is widely used in pharmaceutical applications because of its excellent biocompatibility and biodegradability (16). Various therapeutic agents and drugs can be encapsulated into PLGA microspheres using different methods (17). Additionally, PLGA microspheres can be modified based on their natural properties to achieve the optimum release rate in tissue (18). Therefore, PLGA microspheres are an appropriate emerging carrier for drug and therapeutic agent delivery in nanoparticulate delivery systems.

**Scheme 1:**
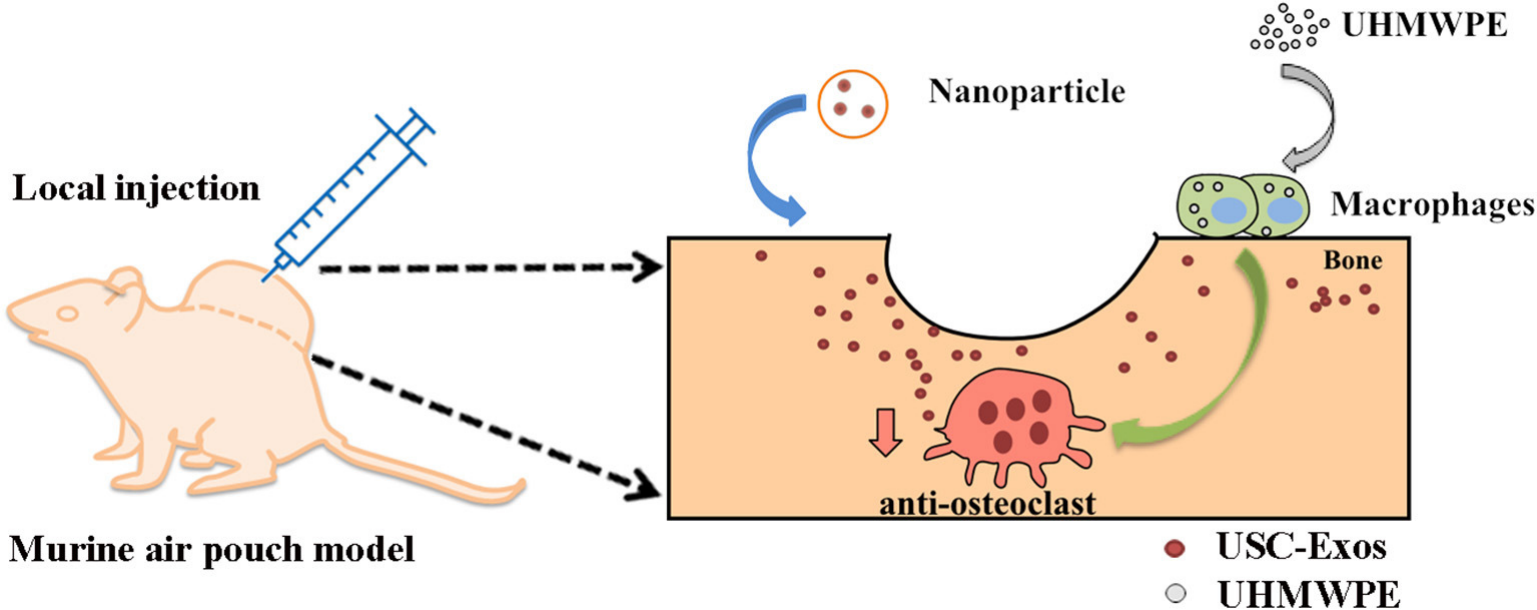
Schematic illustration of therapeutic USC-Exos released from PLGA microspheres for wear particle-induced osteolysis treatment.

According to a previously published article, the high intensity generated by sonication can damage the external structure of exosomes, resulting in exosome reassembly during bioactive nanoparticle formation (19). Based on the former conclusion, this study proposes a sonication strategy using the mechanical double-emulsion method to generate PLGA-Exo-based NPs and tests their characteristics, biodistribution, and therapeutic effects in a murine air pouch model of UHMWPE-induced osteolysis (SCHEME 1).

## Materials and Methods

### Preparation of UHMWPE Microparticles

White micronized UHMWPE particles were purchased from Shamrock TEDA (Tianjin, China). The density of UHMWPE was 0.97 g/cm^3^; thus, the particles floated on the culture medium when added to the medium. Before use, UHMWPE was washed in 75% ethanol and 100% ethanol for 3 days and then sterilized with cobalt 60 γ-radiation. The morphology and size distribution of UHMWPE particles were tested by scanning electron microscopy (SEM) and DLS (Malvern Instruments, Malvern, UK), respectively.

### Identification of the Human USCs

The isolation and identification of USCs were described in detail in a previous study and were performed with a few modifications (13, 14, 20). Briefly, the midstream of human urine from a healthy male donor aged 30 years was collected into a sterile 50-mL tube filled with 500 μL of antibiotic-antimycotic solution (Gibco, USA). The 50 mL of urine was equally distributed into four sterile 15-mL tubes and centrifuged at different speeds, and only 0.2 mL remained in the bottom of the tube. Next, 1 mL of primary medium (Lonza, USA) was added to the tube, and the cell pellet solution was transferred into one well of a 12-well plate. The culture medium was replaced with fresh proliferation medium every 2 or 3 days. The plates were checked daily and cultured until the cells reached 80–90% confluence. P2-6 USCs were used for subsequent experiments.

USCs were induced using specific differentiation medium (Cyagen Biosciences, China) to test their multipotent differentiation potential as described previously (13, 14). A pellet culture technique was used to examine the chondrogenic differentiation of USCs. After coculture for 30 days, alizarin red staining (ARS), oil red O staining, and Alcian blue staining were performed to observe the differentiation potential of USCs. The expression of mesenchymal stem cell (MSC) surface marker proteins on P4 USCs was tested by flow cytometry according to previous articles (13, 14). All antibodies were purchased from BD Biosciences (San Jose, CA, USA).

### Isolation and Characterization of USC-Exos

USC-Exos were isolated from the culture supernatants according to published studies (13, 14). After the cells were cultured with fresh complete proliferation medium containing 10% exosome-free FBS (Shanghai VivaCell Biosciences Ltd., China) for ~48 h, the medium was collected and centrifuged using an Amicon Ultra15 Centrifugal Filter Tube (10 kDa; Millipore). Finally, ~1 mL of ultrafiltration solution was mixed uniformly with ExoQuick-TC Solution (System Biosciences, USA) at a volume ratio of 5:1 by inversion. After centrifugation at 1,500 × g for 30 min, the exosome pellets in the bottom of the tube were resuspended in aseptic PBS based on the volume of the exosome pellets. The protein content of exosomes was measured using the BCA Assay Kit (Multi Sciences LTD., Hangzhou, China). The exosome solution was stored at −80°C until use in subsequent experiments.

The expression of TSG101 (ab125011; Abcam), CD63 (sc-5275; Santa), and calnexin (ab22595; Abcam) in isolated exosomes was determined by western blotting as described previously (14). USC extract was used as a control. The size distribution of USC-Exos was measured by DLS. The morphology of exosomes was observed by TEM (13, 14).

### Determination of the Optimal Concentrations of UHMWPE Particles

RAW 264.7 macrophages (5,000 cells/well) were cocultured with a series of concentrations of UHMWPE particles (0, 0.25, 0.5, 0.75, and 1 mg/mL) for 5 days in 96-well-plates. A Cell Counting Kit-8 kit (NCM Biotech, Suzhou, China) was used to measure cell viability. First, the old medium was completely removed, and fresh DMEM was mixed with the CCK-8 solution at a volume ratio of 10:1 and quickly added to each well. A microplate reader was used to measure the optical density (OD) at 450 nm.

### TUNEL Apoptosis Detection kit

After 48 h of coculture with UHMWPE particles or UHMWPE particles + USC-Exos, RAW 264.7 cells were stained using a TUNEL kit (KeyGen Biotech, China). The nuclei were stained with DAPI (Solarbio, Beijing, China). Next, the stained cells were examined and captured under a fluorescence microscope (Leica, Germany).

### Exosome Uptake Assay

We labeled USC-Exos with PKH26 fluorescent dye (Sigma-Aldrich) to observe whether RAW 264.7 cells can internalize exosomes. Briefly, 1.0 mL of Diluent C was added to 800 μg of exosome solution. Then, 4 μL of PKH26 dye was added to the mixture and cocultured for 5 min at room temperature in the absence of light. The labeled exosomes were then centrifuged at 100,000 rpm for ~25 min at 4°C, and the exosome pellet was suspended in PBS and used for uptake experiments. RAW 264.7 cells were seeded into glass coverslips (Solarbio) and cultured for 24 h until reaching 80–90% density. We then cocultured 25 μL (100 μg) of PKH26-labeled USC-Exos with RAW 264.7 cells in serum-free DMEM culture medium for 4, 12 and 24 h. After the cells were fixed in 4% paraformaldehyde (PFA) for 20 min and permeabilized with 0.1% Triton X-100, the cells were stained with phalloidin Alexa Fluor 488 (A12379; Invitrogen, USA) for 1 h at room temperature. Images were examined and captured under a fluorescence microscope (Leica, Germany).

### Osteoclastic Differentiation Assay

RAW 264.7 cells were seeded into 48-well-plates and cultured overnight in DMEM. Then, the cells were randomized into the following three groups with three wells per group: DMEM + PBS; DMEM + RANKL + PBS; and DMEM + RANKL + USC-Exos. The medium was replaced with fresh DMEM supplemented with 100 ng/mL RANKL (ProteinTech, USA) and 300 μg/mL USC-Exos or an equal volume of vehicle (PBS) after cell adhesion. The culture medium was replaced every other day. Eight days later, the cells were fixed in 4% PFA for 25 min and then stained using a commercial TRAP kit (Sigma-Aldrich). The numbers of TRAP^+^ osteoclasts were counted under a microscope (Leica). The culture medium was collected on the fourth day and evaluated by ELISA [MULTI SCIENCES (LIANKE) BIOTECH, Hangzhou, China]. The levels of osteoclast-related genes were analyzed by RT-qPCR.

### Osteogenic Differentiation Assay

Three-week-old C57BL/6 mice were sacrificed to isolate bone marrow-derived mesenchymal stem cells (BMSCs). Briefly, BMSCs were obtained from the femurs and tibias of the mice. After centrifugation at 1000 rpm for 5 min, the cells were resuspended in α-MEM (Gibco, USA). P1 BMSCs were used in subsequent experiments. BMSCs were seeded into 48-well-plates (1.0 × 10^5^/well). Twenty-four hours later, the old medium was replaced with fresh osteogenesis induction culture medium (Cyagen Biosciences, China) with or without USC-Exos (100 μg/mL). The culture medium was replaced every 2 days. On the 4th day, the old cell medium was collected, and the levels of alkaline phosphatase and calcium were evaluated using commercial kits (Nanjing Jiancheng Bioengineering Institute, China). After induction for 14 days, the cells were stained with ARS solution (Cyagen Biosciences, China). Representative images were examined and captured under an inverted microscope (Leica DMI6000B, Germany).

### Fabrication and Characterization of USC-Exo-Based PLGA NPs

USC-Exos encapsulated into PLGA microspheres were fabricated using a standard mechanical double emulsion technique with modifications (21, 22). First, 100 mg of 0.67 dL/g carboxy-terminated 50:50 PLGA (Jinan Daigang Biomaterial Co., Ltd., Jinan, China) was added to 3 mL of dichloromethane (DCM). Next, the USC-Exo solution was dissolved in 4% cold poly(vinyl alcohol) (PVA) under low-speed shaking for 30 min and slowly injected into DCM containing PLGA for 5 min. The resulting PLGA solution was mixed with the USC-Exos at a weight ratio of 10:1. The mixture was emulsified for 2 min (5 s-0-5 s) using an ultrasonic processor (20 kHz; 130 W; SONIC AND MATERIALS INC., USA). Deionized water (20 mL) was added to the solution and stirred overnight until DCM was completely volatilized. Finally, the mixture was washed three times with PBS at 10,000 rpm for 10 min, and the resulting NP solution was stored at 4°C.

NP size and zeta potential were examined by DLS (Malvern, UK). The morphology of NPs was determined by TEM.

The *in vitro* release of total protein was measured over a period of 12 days, and the cumulative release curve was plotted based on the values. The release profile was assessed using a BCA assay kit (Multi Sciences LTD., Hangzhou, China). Briefly, 2.5 mg of NPs was added to a 1.5-mL sterile tube containing 1 mL of PBS, and then, the mixture was placed in a 37°C incubator (*n* = 3). Next, 20 μL of PBS supernatant was replaced with fresh PBS solution on days 1, 2, 3, 4, 5, 6, 7, 8, 9, 10, 11, and 12 and centrifuged at 14,000 rpm for 10 min. The content of the released protein was detected, and the released percentage was calculated.

The *in vitro* biodegradability of NPs was evaluated by assessing the changes in the size and zeta potential by DLS (Malvern, UK) at room temperature and 4°C.

### Cellular Uptake Assay of NPs

To investigate the cellular uptake phenomenon of PLGA-based NPs, NPs (10 mg/mL PLGA) fabricated from PLGA microspheres were labeled with DiI (Beyotime, Shanghai, China), and USC-Exos were labeled with DiO (Yeasen, Shanghai, China) and then incubated with RAW 264.7 cells (1 × 10^4^ cells) in 6-well-plates at 37°C for 12 and 24 h. Before incubation, the cells were cultured in the wells overnight to recover. After incubation, cell nuclei were stained with DAPI (Solarbio, Beijing, China). Cell images were evaluated on a fluorescence microscope (Zeiss LSM 410, Germany).

### *In vitro* Evaluation of Cytotoxicity

After 24 h of incubation in high-glucose DMEM, RAW 264.7 cells were incubated with different concentrations of NPs (0.02–0.10 mg/mL PLGA) for 24 h. A commercial CCK-8 kit (NCM Biotech, China) was used to evaluate cell proliferation.

### Murine Pouch Model of Bone Resorption and Treatment

Animal studies were approved by the Laboratory Animal Research Center of Central South University (No. 2019030499). Forty-eight female BALB/c mice (8–10 weeks old) were purchased in total. The detailed procedure to establish the mouse air pouch model was performed according to previous articles with slight modifications (23). Briefly, 2 × 2 cm^2^ dorsal skin was disinfected with alcohol and shaved to expose the pouch site. One milliliter of sterile air was injected at a single site using a 25-gauge needle and 2-mL syringe every other day for 7 days to maintain the air pouch. Seven days later, mice that successfully established air pouches were anaesthetized. The congeneric mice were sacrificed as calvarial bone donors (sixteen mice in total; one donor provided a cranial bone implant for two recipients). Thereafter, a 0.5-cm-long incision was made along the skin of the pouch, and one piece of ~0.5 × 0.5-cm calvarial bone from a donor mouse was inserted into the pouch using forceps. Next, 20 mg of UHMWPE was added to the air pouch to stimulate the inflammatory reaction and bone osteolysis. 4-0 sutures were used to close the skin incision. Twenty-four hours postoperation, 32 mice were randomly divided into the following four groups of 8 to receive local injections into the pouches: the vehicle control group (sterile PBS); the UHMWPE + PBS group (200 μL); the UHMWPE + USC-Exos group (200 μL, 200 μg); and the UHMWPE + NPs group (200 μL, 200 μg). The mice were sacrificed on day 21, and both the pouch membrane and implanted bone were harvested (*n* = 8 per group) and fixed in 10% PFA for paraffin embedding.

### Fluorescent Imaging for the Biodistribution of NPs

Equal volumes of USC-Exos (200 μL, 200 μg) and NPs (200 μL, 200 μg) loaded with DiI were injected into murine air pouches. Equal volumes of PBS (200 μL) were used as the control. Fluorescent images were obtained after day(s) 1, 3, 5, 7, 9, 11, and 14 using a fluorescence tomography imaging system (Bruker, USA). Fourteen days after the injection, major organs were collected for biodistribution studies.

### Micro-CT Analysis

High-resolution micro-CT (PerkinElmer, Aartselaar, USA) was used to quantitatively analyze the implanted bone mass. The calvariums (*n* = 7 or 8) were scanned at 36 mm FOV, with 72 μm pixel size and 90 kV and 80 μA X-ray energy. After the effective data were obtained, the two-dimensional images were reconstructed into three-dimensional images, which were then carefully observed to determine the erosion surface. The bone mineral density (BMD) and bone volume fraction (bone volume/total volume, BV/TV) were calculated to assess bone loss using auxiliary software (Analyze 12.0, USA).

### Histological Staining and Analysis

After micro-CT scanning, formalin-fixed implanted bones were decalcified in 10% EDTA for 7 days. Next, H&E staining was used to stain tissue sections (6 μm) to observe pouch membrane inflammation and bone erosion. A histochemical TRAP kit (Sigma-Aldrich, St. Louis, MO, USA) was used to evaluate osteoclast-like cells in the implanted calvariums, and the numbers of osteoclasts over the adjacent bone surface (N/mm) were calculated. A light microscope (Olympus CX31; Olympus Optical Co., Tokyo, Japan) was used to examine and capture images of the stained sections. In total, six points in each section were used to measure pouch membrane thickness as previously described (23). The images were analyzed using Image-Pro Plus.

### Safety Examination

Blood from each group was sampled after anesthesia and analyzed using routine blood tests to assess immunogenicity. H&E staining of the main organs was used to evaluate the safety of the NPs and USC-Exos.

### RT-qPCR Analysis

Total cellular RNA from each sample was extracted. The 2^−ΔΔCT^ method was used to calculate gene expression, and GAPDH was used as the internal control. The primer sequences were as follows: Trap: forward, 5′*-TGGTCCAGGAGCTTAACTGC-*3′, and reverse, 5′*-GTCAGGAGTGGGAGCCATATG-*3′; Mmp9: forward, 5′*-ACCCGAAGCGGACATT-*3′, and reverse, 5′*-GGCATCTCCCTGAACG-*3′; Gapdh: forward, 5′*-ATCCCATCACCATCTTCC-*3′, and reverse, 5′*-GAGTCCTTCCACGATACCA-*3′.

### Statistical Analysis

All data are shown as the means ± standard deviation (SD). Statistical analysis of the data was performed using SPSS 19.0 software (SPSS, Inc., Chicago, IL, USA). Analyses were performed using one-way analysis of variance (ANOVA), and Tukey's *post-hoc* test was used. *P* < 0.05 was considered significant.

## Results

### UHMWPE Particle Characterization

**Figure 1:**
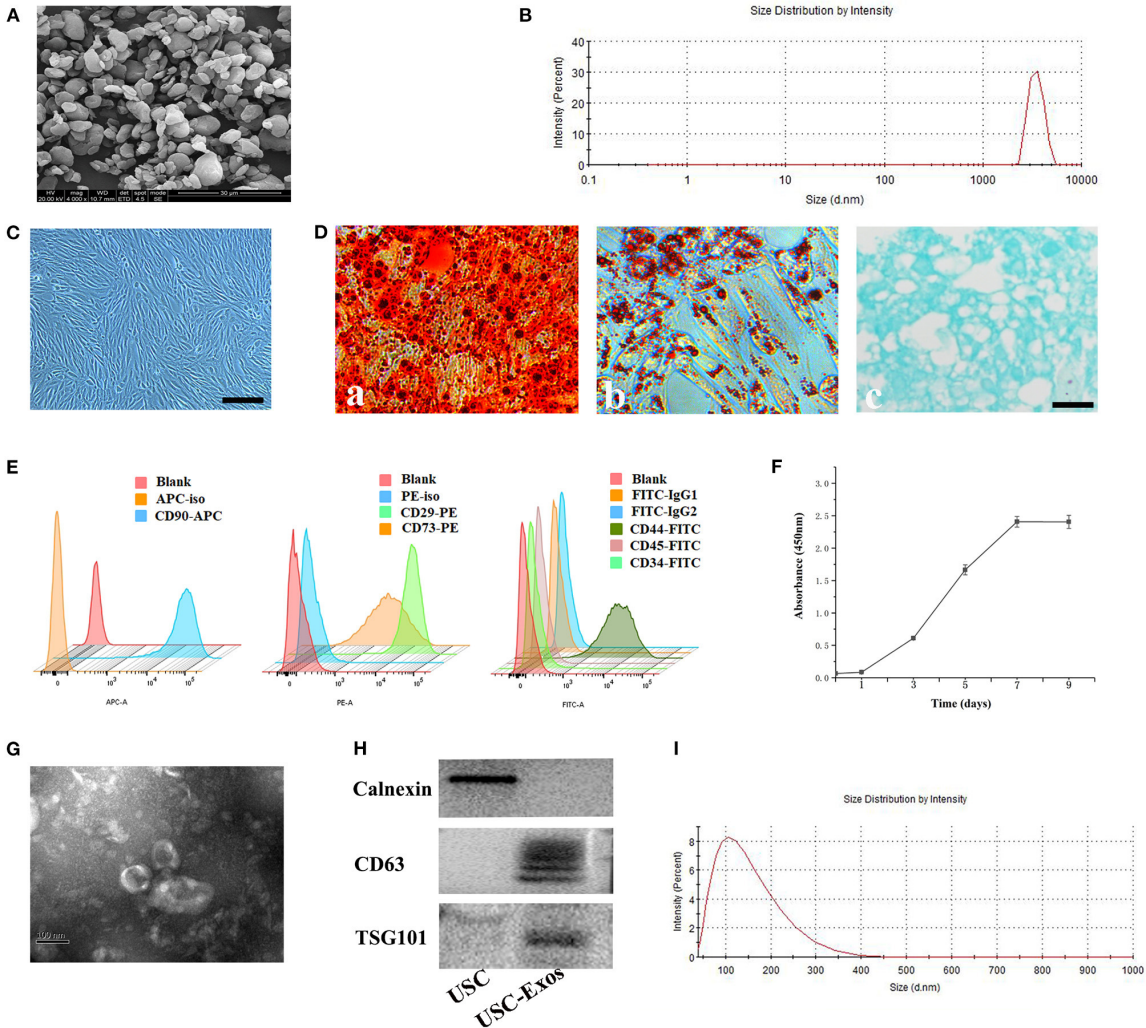
Characterization of UHMWPE microparticles, USCs and USC-Exos. **(A)** SEM image of UHMWPE microparticles (scale bar, 20 μm). **(B)** UHMWPE was analyzed for size distribution using DLS. **(C)** USCs had a spindle-like morphology. Scale bar, 30 μm. After culture in osteogenic, adipogenic or chondrogenic induction medium, USCs could differentiate into osteoblasts **(D**-a; scale bar, 30 μm**)**, adipocytes **(D**-b; scale bar, 30 μm**)** or chondrocytes **(D-**c; scale bar, 30 μm**)**. **(E)** Flow cytometry analysis of the typical surface markers in USC-Exos. **(F)** USC growth curve. **(G)** Morphology of USC-Exos under transmission electron microscopy. Scale bar, 100 nm. **(H)** Detection of exosome surface markers in USC-Exos by western blotting. **(I)** Size distribution of USC-Exos calculated by DLS. The data are expressed as the mean ± standard deviation. The experiment was repeated three times independently.

SEM and DLS were used to assess the morphology and size distribution of UHMWPE particles (Figures 1A,B). The classical morphology of UHMWPE particles is characterized by an irregular shape and rough surface (Figure 1A). Regarding size distributions, more than 90% of the particles were in the size range of 0.5–5 μm (Figure 1B).

### Characterization of USCs and USC-Exos

The morphology of human urine cells at different times after collection is shown in Supplementary Figure 1. The adherent USCs possessed a fibroblast-like morphology (Figure 1C). The USCs could differentiate into osteogenic, chondrogenic and adipogenic lineages, as detected by ARS staining, toluidine blue staining and oil red O staining, respectively (Figure 1D). The USCs were characterized by flow cytometry for common MSC markers (Figure 1E). Additionally, the CCK-8 assay showed that the USCs grew rapidly from day 3 to 7, and growth slowed 7 days later (Figure 1F). SEM, DLS, and western blotting were used to evaluate the isolated extracellular particles. First, TEM showed that the extracellular particles possessed a cup-shaped morphology with a double-layered membrane structure (Figure 1G). Second, western blot analysis demonstrated that the extracellular particles secreted by USCs were positive for CD63 and TSG101 but negative for calnexin (Figure 1H). Finally, the size distribution was measured using DLS, demonstrating that these particles mainly ranged from 50 to 150 nm in size (Figure 1I). Together, these data demonstrated that the extracellular particles released from USCs in the study were exosomes.

### Cell Proliferation and Apoptosis Analysis in RAW 264.7 Cells After UHMWPE or USC-Exo Exposure

**Figure 2:**
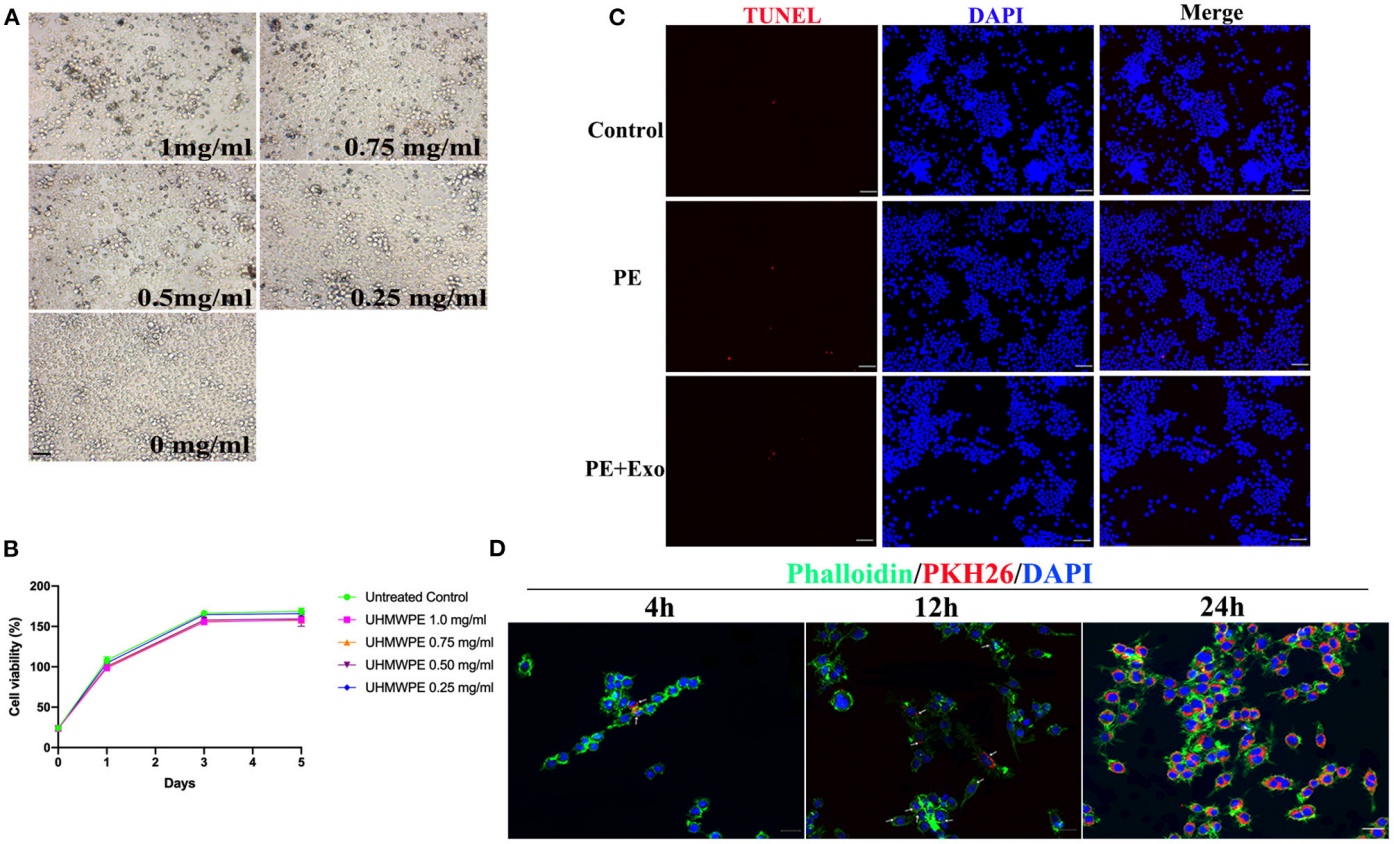
Effects of UHMWPE and UHMWPE + USC-Exos on the viability of RAW 264.7. **(A)** Various concentrations of UHMWPE particles (dark dots) were internalized into the cells. Scale bar, 30 μm. **(B)** RAW 264.7 proliferation was measured after coculturing with UHMWPE. **(C)** Representative images of TUNEL staining in RAW 264.7 cells exposed to 1 mg/mL particles for 48 h. Scale bar, 50 μm. **(D)** Representative immunofluorescence images showing the internalization of PKH26-labeled USC-Exos (red) by RAW 264.7 cells stained with phalloidin (green) at 4, 12, and 24 h. Cell nuclei were stained with DAPI (blue). White arrows indicate exosomes (red). Scale bar, 20 μm. The data are expressed as the mean ± standard deviation. The experiment was repeated three times independently.

To determine the optimal concentration for UHMWPE particles, different concentrations of UHMWPE particles were cultured with RAW 264.7 cells. RAW 264.7 cells phagocytosed UHMWPE particles (dark dots) (Figure 2A); 1 mg/mL UHMWPE particles (dark dots) were obviously internalized into the cells, and the particles did not severely influence cell viability (Figure 2B). Thus, we selected 1 mg/mL UHMWPE particles as the optimal concentration for use in downstream experiments. The TUNEL assay was performed to observe RAW 264.7 cell apoptosis after coculturing with UHMWPE particles or USC-Exos. Figure 2C shows representative images of TUNEL-positive apoptotic cells exposed to UHMWPE or UHMWPE + USC-Exos compared with the nonparticle-containing medium. No obvious differences were found in the number of apoptotic cells among the polyethylene particle and polyethylene particle + USC-Exo groups.

### Internalization of Exosomes by RAW 264.7 Cells

We cocultured PKH26-labeled exosomes with RAW 264.7 cells for 4, 12, and 24 h to observe whether exosomes could access the cytoplasm of RAW 264.7 cells. Fluorescence microscopy images (Figure 2D) showed that PKH26-labeled USC-Exos (red dots) were gradually endocytosed into RAW 264.7 cells from 4 to 24 h. Twenty-four hours postcoculture, PKH26-labeled exosomes were largely endocytosed and spread in the cytoplasm.

### Fabrication and Characterization of NPs

**Figure 3:**
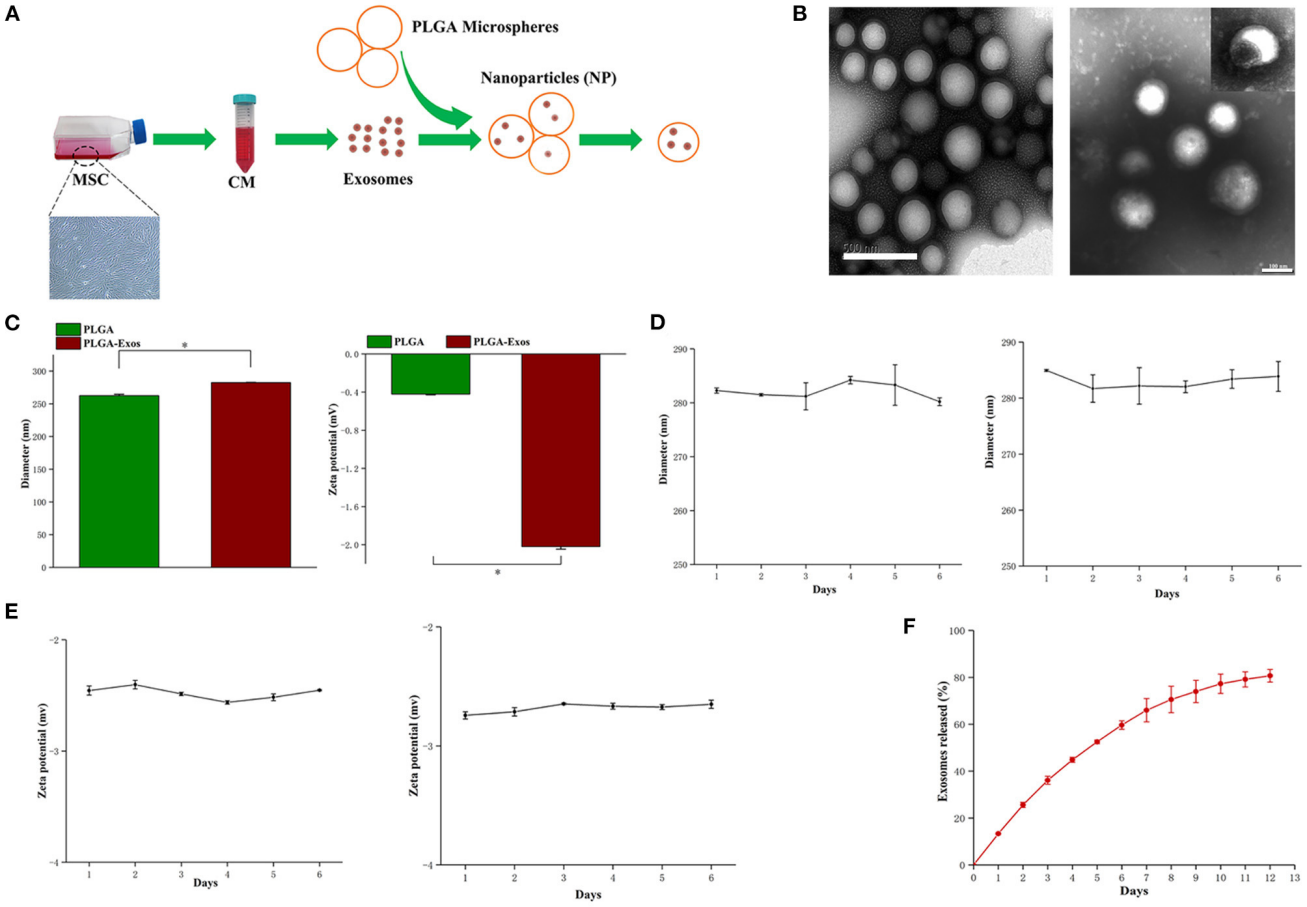
Physiochemical and biological properties of NPs. **(A)** Schematic illustration of the preparation of NPs. **(B)** TEM image of PLGA microspheres and NPs fabricated with the mechanical double-emulsion method. Scale bar, 500 nm (left) and 100 nm (right). **(C)** Diameters (nm, left) and zeta potentials (mV, right) of PLGA microspheres and NPs measured by DLS (*n* = 3). *P < 0.05. **(D)** Size change of NPs after storage at room temperature (left) and at 4°C (right) (*n* = 3 for each time point). **(E)** Zeta potential of NPs after storage at room temperature (left) and at 4°C (right) (*n* = 3 for each time point). **(F)** Quantitative analyses of the releases of exosomes from NPs over time. The data are expressed as the mean ± standard deviation. Statistical analysis was performed using one-way analysis of variance (ANOVA), and Tukey's *post-hoc* test was used. The experiment was repeated three times independently.

The schematic design of NPs is summarized in Figure 3A. First, USC-Exos were incorporated into PLGA microspheres to form NPs. TEM imaging confirmed the empty PLGA microspheres and NPs (Figure 3B). A TEM image of NPs showed a spherical shell, and USC-Exos were dispersed in the core of PLGA microspheres (Figure 3B), similar to previous reports (22). NanoSight characterization revealed that the size of the NPs was ~282 ± 0.4 nm, which was slightly greater than that of empty PLGA particles (262 ± 2.0 nm). The zeta potential of the NPs was −2.02 ± 0.03 mV (Figure 3C). Thereafter, the stability of the NPs was measured by observing the changes in size and zeta potential over time at room temperature and 4°C. The NPs remained stable for more than 6 days, with a negligible increase in size and zeta potential (Figures 3D,E). The release profiles were determined over 12 days (Figure 3F).

### *In vitro* Bioactivity of USC-Exos and NPs

**Figure 4:**
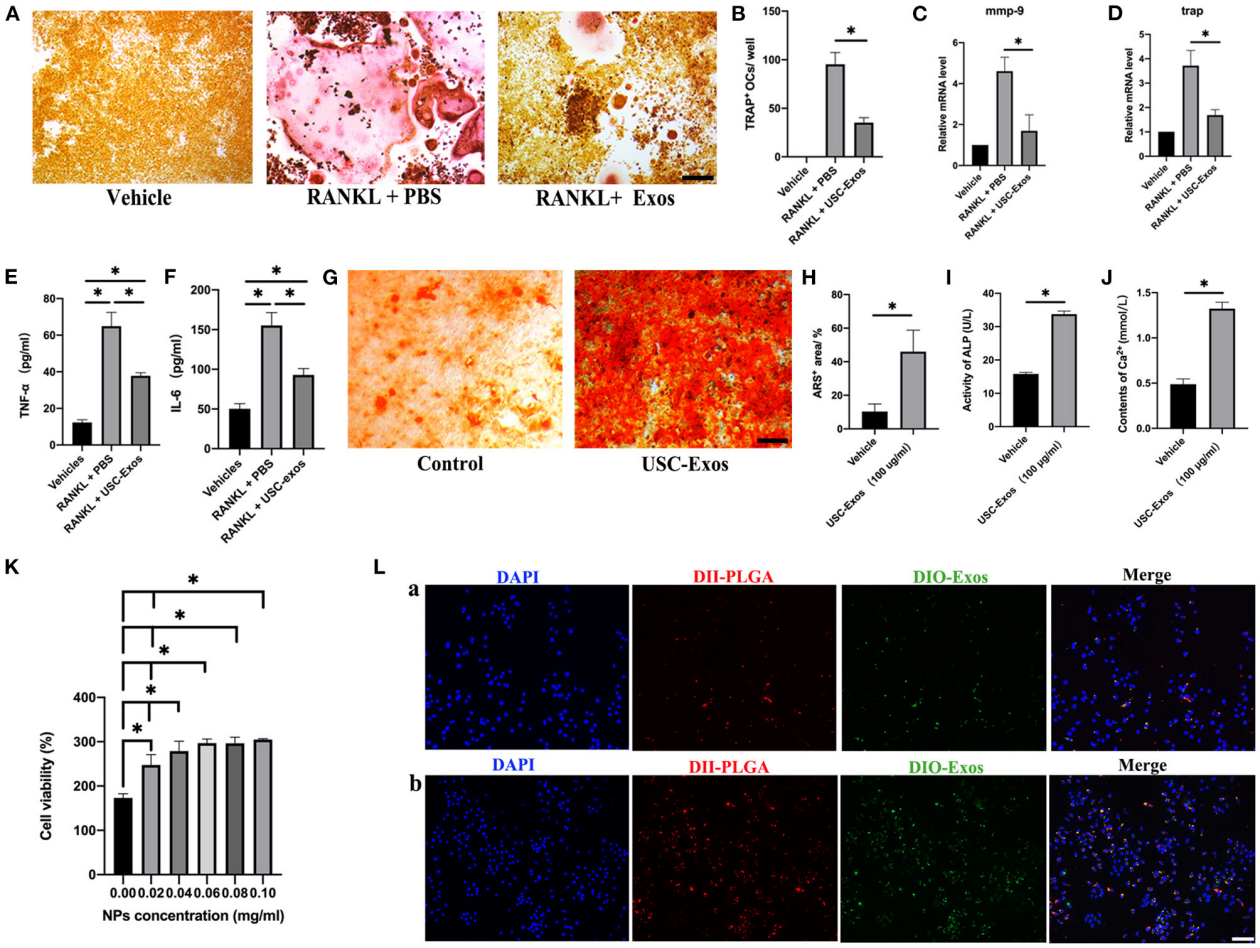
*In vitro* bioactivity of USC-Exos and NPs. **(A)** Osteoclast differentiation of RAW 264.7 cells from different groups determined by TRAP staining. Scale bar, 30 μm. **(B)** The numbers of TRAP^+^ multinucleated (>3 nuclei) osteoclasts were counted. *n* = 3 per group. *P < 0.05. The expression levels of Mmp9 **(C)** and Trap **(D)** were assessed by RT-qPCR analyses. *n* = 3 per group. The concentrations of TNF-α **(E)** and IL-6 **(F)** were determined by ELISA. *n* = 3 per group. *P < 0.01. **(G)** Alizarin red S (ARS) staining of BMSCs from different groups under osteogenic inductive conditions. Scale bars, 30 μm. Quantitative analyses of ARS^+^ area **(H)**, ALP **(I)** and Ca^2+^ **(J)** in conditioned media from BMSCs receiving different treatments. *n* = 3 per group. *P < 0.05. **(K)** Percentages of proliferating RAW 264.7 cells under various NP concentration treatments (*n* = 3 for each group). **(L)** Macrophage uptake of NPs after 12 h (a) and 24 h (b) of coincubation (*n* = 3 for each group). Scale bar, 30 μm. The data are expressed as the mean ± standard deviation. Statistical analysis was performed using one-way analysis of variance (ANOVA), and Tukey's *post-hoc* test was used. The experiment was repeated three times independently.

RAW264.7 cells were stimulated with RANKL and cocultured with USC-Exos or PBS for 7 days to observe the influence of USC-Exos on osteoclastogenesis. TRAP staining images showed that RAW 264.7 cells induced by RANKL differentiated into many TRAP^+^ osteoclasts, whereas USC-Exo treatment suppressed osteoclast formation (Figures 4A,B). RT-qPCR analysis demonstrated that the RANKL-induced groups had high expression levels of Mmp9 and Trap mRNA compared with the vehicle group, but the effect was strongly inhibited by USC-Exos (Figures 4C,D). The values of TNF-α and IL-6 in the CM from the RANKL + PBS group were higher than those in the CM from the other two groups, and USC-Exo intervention obviously decreased the production of TNF-α and IL-6, as assessed by ELISA (Figures 4E,F). BMSCs were cultured in osteogenic induction medium containing USC-Exos or an equal volume of PBS to observe the effects of USC-Exos on the formation of osteoblasts. ARS staining showed that USC-Exos obviously enhanced the formation of calcium nodules (Figures 4G–J).

We cocultured RAW 264.7 cells using different concentrations of NPs *in vitro*. The CCK-8 cell proliferation assay revealed that with increasing concentrations of NPs, the viability of macrophages increased, indicating that NPs were noncytotoxic (Figure 4K). We also designed dual fluorescent NPs by labeling PLGA with DiI (red fluorescence) and USC-Exos with DiO (green fluorescence) to explore the internalization mechanism of these NPs. We cultured RAW 264.7 cells using these biomimetic NPs for 12 h (Figure 4La, Supplementary Figure 2) and 24 h (Figure 4Lb, Supplementary Figure 2). RAW 264.7 cells cocultured with fluorescently labeled NPs showed obvious internalization of DiI and DiO into the cytoplasm, demonstrating the entry of NPs into cells by endocytosis (Figure 4L). Additionally, the locations of the PLGA microspheres and USC-Exos were identical, suggesting that the USC-Exos were successfully encapsulated into the PLGA microspheres during the synthesis process, and the fluorescence signal intensity was higher at 24 h.

### Biodistribution of NPs in Mice With UHMWPE-Induced Osteoclastic Bone Resorption

**Figure 5:**
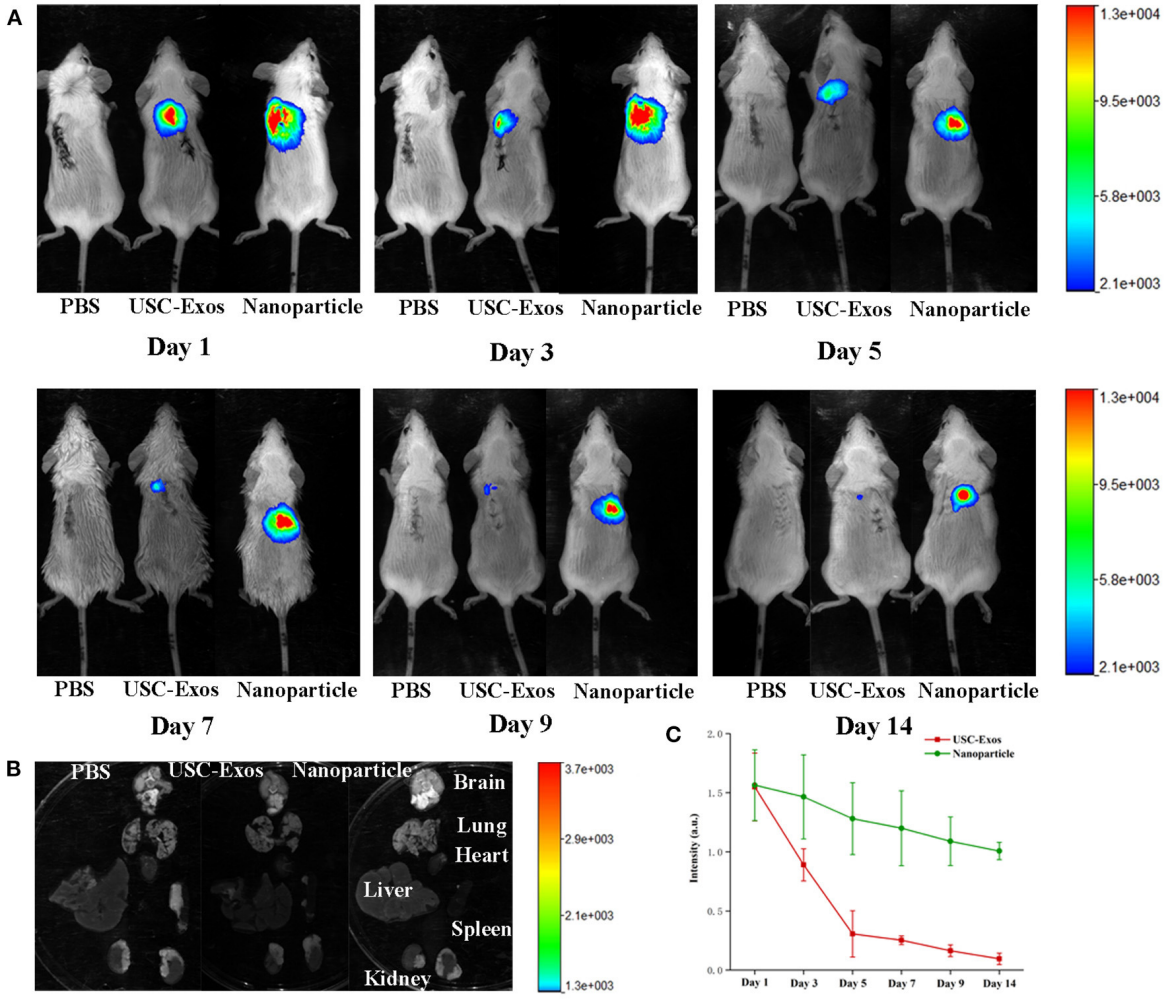
NP biodistribution in the air pouch of mice by fluorescence imaging *in vivo*. **(A)** DiR-labeled USC-Exos and NPs were injected into the air pouch of the mice, and then these mice were imaged by IVIS at the indicated times. **(B)** Fourteen days after injection, the mice were sacrificed, and the organs were removed for imaging. **(C)** Quantitative fluorescent intensities shown in **(A)**, *n* = 3 per group. The data are expressed as the mean ± standard deviation. Every group contained three species independently.

To investigate the biodistribution of NPs, we first established a mouse bone resorption model and then injected NPs and USC-Exos labeled with DiR into the air pouches of mice. PBS was injected as a control. The mice were imaged using IVIS equipment at the indicated times. The fluorescence intensity gradually decreased within 2 weeks, but fluorescence was still detected in the local air pouch at the end time (Figure 5A). Higher fluorescence was observed for NPs than USC-Exos at the indicated time, and the fluorescence signal of NPs was sustained at a high level at the endpoint (Figures 5A,C). Next, the major organs were harvested at the endpoint, and the fluorescence signal could not be detected in the brain, lung, heart, liver, spleen, or kidneys (Figure 5B). This finding indicates that NP translation and efficacy may be hindered by the air pouch. Because the DiR-labeled USC-Exos and NPs were maintained for ~2 weeks, we chose to sacrifice the animals on day 21.

### NP Therapy Prevents UHMWPE-Induced Osteoclastic Bone Resorption

**Figure 6:**
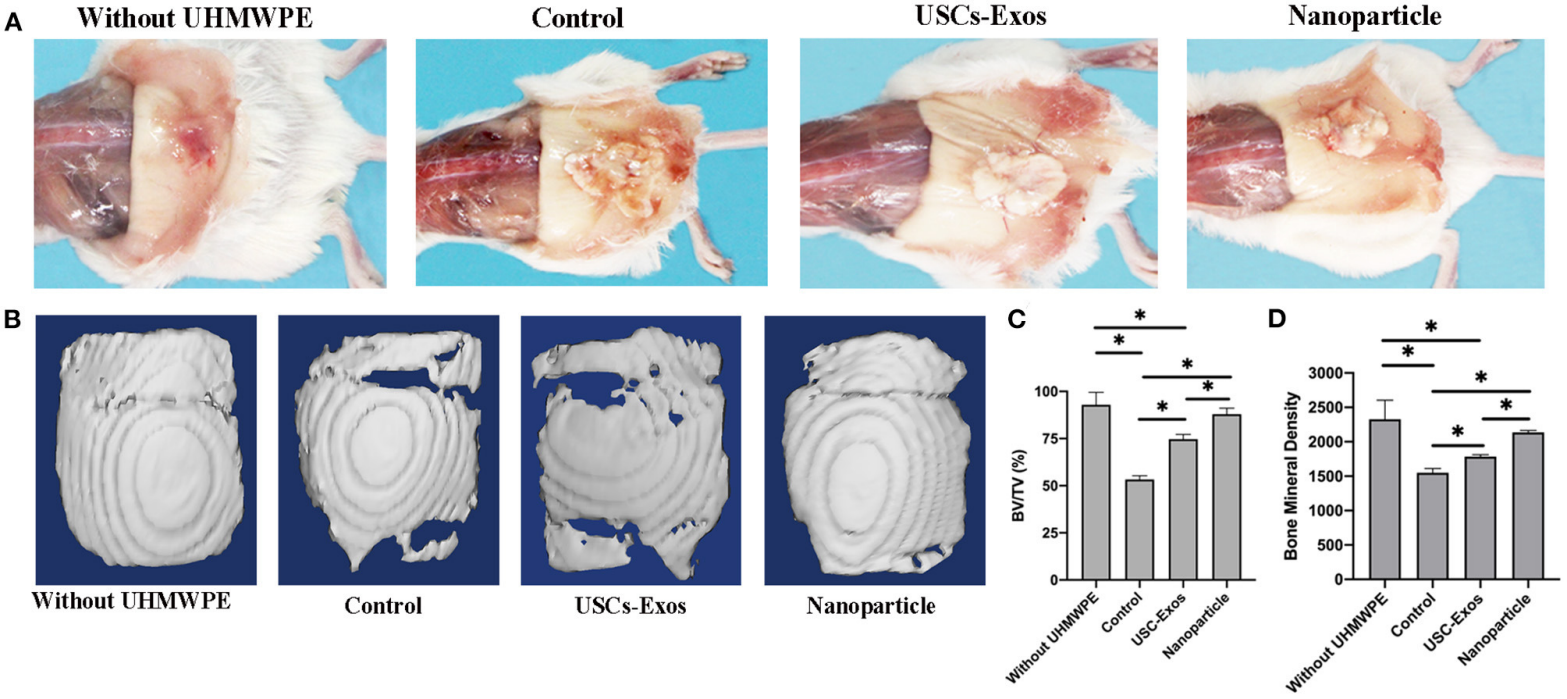
Representative images of pouch tissues and micro-CT evaluation of the implanted bones. **(A)** Typical macroscopic images of air pouches and the implanted calvarial bone dissected from mice. **(B)** Examples of micro-CT surface images of the implanted calvarial bone among groups. *n* = 4 or 5 per group. **(C)** Quantification of bone fraction volume/total volume in **(B)**. *n* = 4 or 5 per group. *P < 0.05. **(D)** Quantification of bone mineral densities in **(B)**. *n* = 4 or 5 per group. *P < 0.05. The data are expressed as the mean ± standard deviation. Statistical analysis was performed using one-way analysis of variance (ANOVA), and Tukey's *post-hoc* test was used. Every group contained 4 or 5 species independently.

The cranial bone implants were well-tolerated in the air pouch by recipient mice for up to 21 days with no noticeable skin infection. UHMWPE particles induced inflammation in the pouch membrane and implant bones, a finding that was consistent with previous findings (23). The gross pathology shown in Figure 6A demonstrated that air pouches supplemented with UHMWPE particles caused a serious inflammatory reaction in mice compared with pouches without UHMWPE particles. USC-Exo and NP treatment alleviated the inflammatory response induced by UHMWPE particles, as shown by the decreased irritant tissue proliferation around the bone in the air pouch.

Micro-CT scanning was used to assess the influence of NPs on the implant cranial bone mass in mice. Micro-CT analysis indicated that the implanted calvarial bones without UHMWPE introduction maintained normal bone mass, and the osteolysis mouse model with UHMWPE introduction demonstrated osteolytic phenotypes, as indicated by significantly reduced BV/TV and BMD values (Figures 6B–D). Additionally, with the loading of exosomes into PLGA microspheres, the BV/TV and BMD values were higher than those of pure exosomes, indicating that the NPs inhibited osteoclastic activity to preserve bone microstructure through sustained release of exosomes (Figures 6C,D).

**Figure 7:**
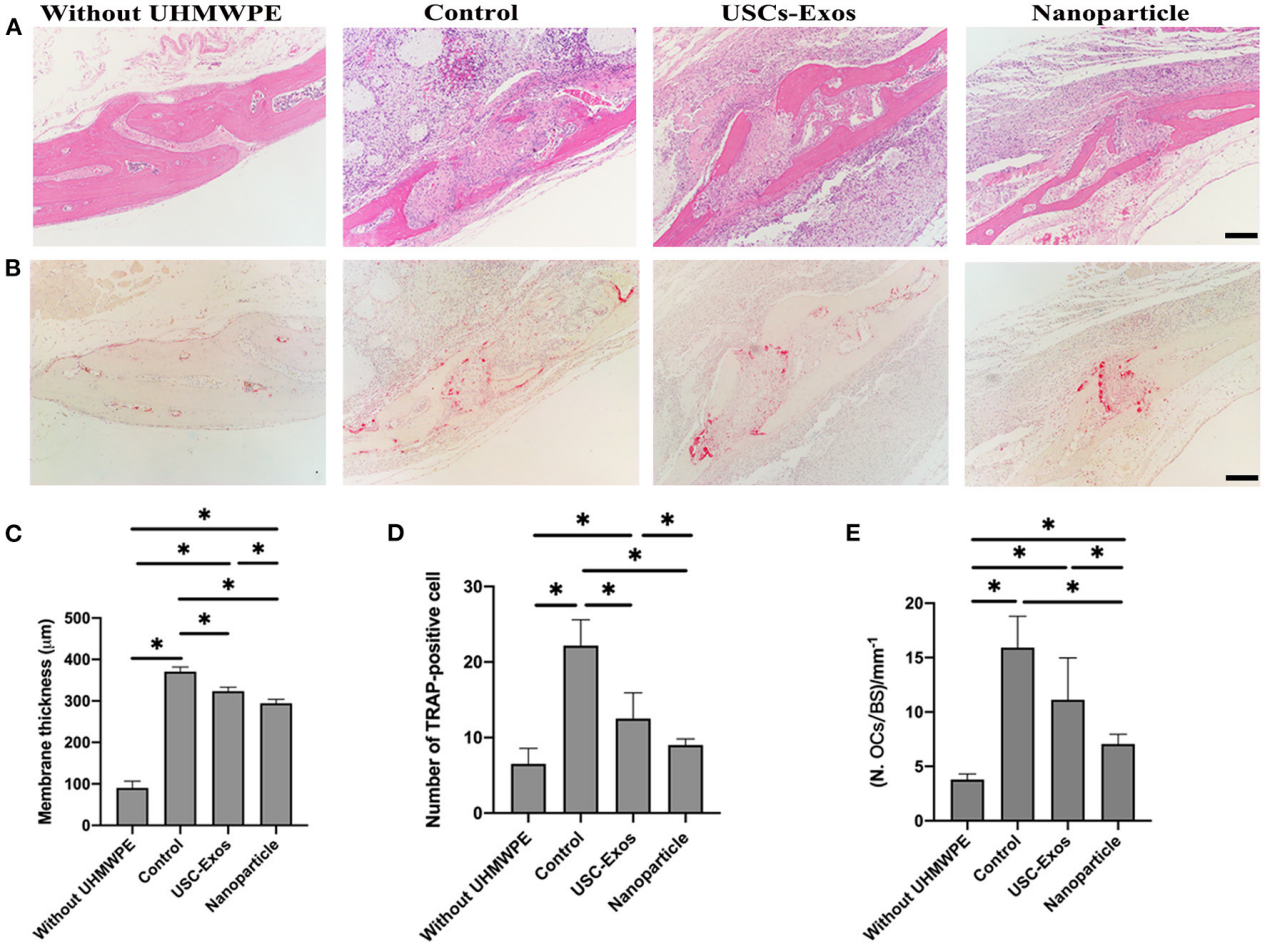
Therapeutic effect of NPs in mice with particulate polyethylene-induced osteolysis. **(A)** Representative images of H&E staining in implanted bones posttreatment. Scale bar, 100 μm. *n* = 4 or 5 per group. **(B)** Representative images of TRAP staining in implanted bones posttreatment. Scale bar, 100 μm. *n* = 4 or 5 per group. **(C)** Quantification of membrane thickness around the implanted bones in **(A)**. *n* = 3 or 4 per group. *P < 0.05. Quantification of the number of osteoclasts (OCs, **D**) and OCs/BS **(E)** on the trabecular bone surface in **(B)**. *n* = 4 or 5 per group. *P < 0.05. The data are expressed as the mean ± standard deviation. Statistical analysis was performed using one-way analysis of variance (ANOVA), and Tukey's *post-hoc* test was used. Every group contained 4 or 5 species independently.

Next, we performed H&E staining of the implanted bone to observe the changing status of osteolysis during treatment (Figures 7A,C). H&E staining of air pouch membranes and the implanted bones differed among the groups (Figure 7A). The presence of UHMWPE particles induced local inflammatory reactions, as shown by the rises in membrane thickness and bone absorption compared with those in the nonparticle-containing groups. These effects were significantly alleviated by NP and USC-Exo treatment (Figures 7A,C). Inflammatory cells eroded into the bone, resulting in the erosion of bone in contact with pouch membranes. Higher membrane thickness around the bone and absorption of the bone were observed in the UHMWPE + PBS-treated groups; however, the NP and USC-Exo treatment groups showed alleviated results. The membrane thickness data were also consistent with the results of H&E staining, which showed the thinnest membrane in the NP group, followed by the exosome treatment group and the control group (Figure 7C).

TRAP staining was used to observe whether NPs and USC-Exos could suppress UHMWPE-induced osteolysis. The TRAP staining results of the implanted bone are shown in Figure 7B. Many osteoclasts were observed in the UHMWPE + PBS group, and the number of osteoclasts was higher than that in the other three groups (i.e., the exosome, NP and control groups) (Figures 7B,D,E). The control group without the introduction of UHMWPE showed fewer osteoclasts than the other three groups. However, an obviously minimal number of TRAP^+^ cells was observed in the NP treatment group (Figures 7D,E). These data were also consistent with H&E staining, in which the NP group showed lower membrane thickness and bone absorption than the UHMWPE + PBS and USC-Exo groups.

### Safety of NPs

**Figure 8:**
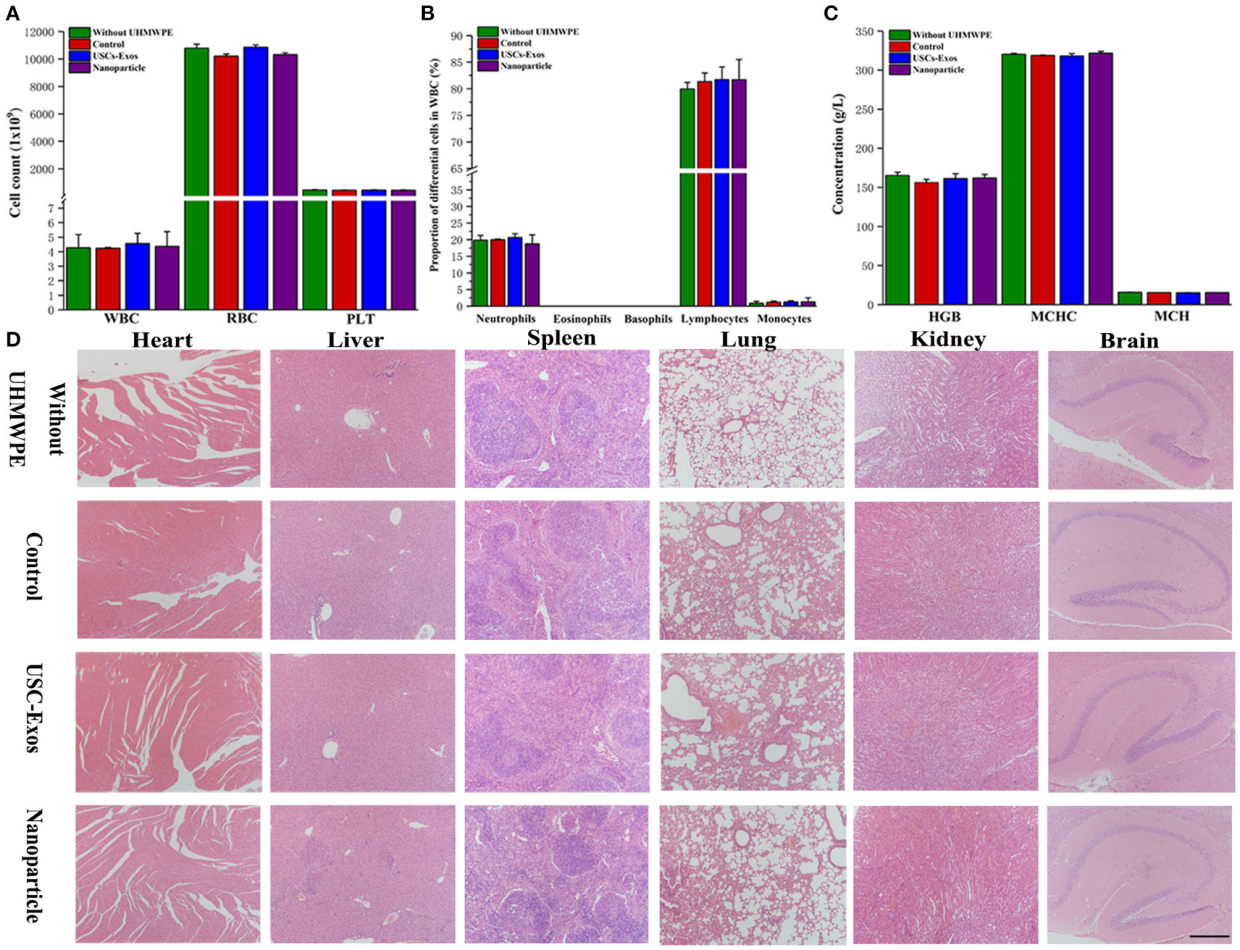
Biocompatibility and safety of NP treatment. **(A)** White blood cells, red blood cells and platelets were tested in the groups at day 21. *n* = 4 or 5 per group. **(B)** Proportions of differential white blood cells after NP treatment. *n* = 4 or 5 per group. **(C)** Routine blood examination parameters after NP treatment. *n* = 4 or 5 per group. **(D)** H&E staining of major organs in the groups; scale bar, 100 μm. *n* = 4 per group. The data are expressed as the mean ± standard deviation. Every group contained 4 or 5 species independently.

Currently, the major concerns regarding the *in vivo* application of NPs and USC-Exos are in regard to their safety. The red blood cell test results showed no significant differences between the different groups (Figures 8A–C), and H&E staining of the major organs showed no noticeable histopathological changes in the groups (Figure 8D).

## Discussion

Joint instability, infection and patellofemoral problems are the main reasons for reoperation in the early years after primary total joint replacement; however, one of the most common and critical late complications leading to arthroplasty failure is aseptic loosening caused by periprosthetic osteolysis (24). Periprosthetic osteolysis initially begins with the production of wear debris *via* the mechanical wear of the prosthesis, which gradually accumulates at the bone-implant interface, playing an increasingly important role in inducing osteolysis (25). Aseptic loosening is a complex issue that requires thorough evaluation and a detailed preoperative plan. Prosthetic products have a fundamental influence on biological parameters in the bone-prosthesis environment. UHMWPE particles produced from prostheses have been increasingly considered to have the ability to induce inflammatory reactions and osteolysis (26–28), which would directly cause the failure of total joint arthroplasty (TJA). Revision TJA raises concerns about remaining bone defects and joint instability. Additionally, the revision operation has been associated with high patient morbidity and great financial burden for the health care system (29, 30).

In this study, we used readily available and unlimited human urine samples to obtain stem cells and isolate exosomes from these cells. MSC-derived exosomes contain bioactive components, such as RNA and proteins, which can be delivered to recipient cells to regulate intercellular interactions, thereby promoting bone regeneration (12, 14, 31). USC-derived exosomes also contain a range of biological proteins, including DMBT1 to promote angiogenesis and CTHRC1 and OPG to promote osteogenesis and inhibit osteoclastogenesis, as reported in a previous study (13, 14). Therefore, exosomes represent great promise in regenerative medicine and drug delivery systems; however, inherent obstacles, such as their rapid elimination by macrophages and long-term preservation of their bioactive function in the blood circulation, must be overcome (32).

In a drug sustained release system, the main requirement for the successful delivery of various therapeutic drugs or agents is to preserve their bioactive components during the process of encapsulation and release. Hence, a strategy that allows for a controlled release and maintains the biological properties of stem cell-derived exosomes is needed to effectively promote tissue repair and regeneration. Here, we utilized the double-emulsion method to fabricate designer NPs with small sizes and core-shell structures by loading PLGA particles with USC-Exos to preserve the biological properties of USC-Exos. Additionally, the TEM image of the NPs (Figure 3B) and the double-labeled dye results of the NPs (Figure 4L) showed that USC-Exos could be encapsulated into biodegradable PLGA microspheres *via* ultrasonic emulsification, which can maintain their cargo and biological activity during the manufacturing process. Our study demonstrates that the size of NPs is 280 nm, which is smaller than that previously reported (22), and the zeta potential is slightly negative and can support long-term storage without significant coaggregation. USC-Exos were released only as the PLGA microspheres degenerated, and our results showed that USC-Exos could be controlled for release from NPs for 12 days *in vitro* (Figure 3F) and could maintain sustained release for ~14 days *in vivo* (Figure 5A). Designer NPs are also easily injected *in vivo*, allow the local and sustained release of USC-Exos and modulate downstream cell activities in the same way as management *in vitro* (33). We provide evidence that local air pouch injection of synthetic NPs with therapeutic USC-Exo release could suppress implanted calvarial bone absorption in a murine osteolysis model compared with the injection of exosomes alone by inhibiting osteoclastogenesis. The efficient amelioration of bone absorption was likely due to the sustained release of bioactive exosomes encapsulated into the PLGA microspheres. The NP group showed evident advantages in the study compared with the exosome group because NPs efficiently preserved the biological activity of exosomes and released them to mediate bone protection *in vivo*; nevertheless, a single local injection of USC-Exos in the air pouch could cause the USC-Exos to degenerate quickly or be phagocytized by the macrophages. Thus, we believe that biological techniques that use exosomes as sustained release drugs may have considerable value and clinical advantages in the treatment of osteolysis.

At present, many methods and excellent biomaterials (e.g., scaffolds, hydrogels, or PLGA microspheres) have been reported to deliver exosomes to optimize *in vivo* applications and achieve sustained release (22, 33–37), and significant effects have been obtained. Recently, researchers have reported advanced methods to deliver exosomes *via* microfluidic devices (33); however, the purchase and utilization of instruments and equipment may limit their large-scale application. Other researchers have utilized physical cross linking to incorporate exosomes and fabricate delivery systems (34–37). In our study, we attempted to fabricate designer NPs by loading PLGA nanoparticles with USC-Exos *via* the double-emulsion method; our results showed that the method successfully loaded the exosomes. However, the lack of data to calculate the drug loading capacity and encapsulation efficiency is a limitation of the study. Our study also lacks information about the underlying mechanisms of exosomes in the treatment process, which requires further exploration in our follow-up experiments. As previously reported, exosomes contain many functional proteins and miRNAs (9, 12–14, 31, 36), and the substantial enrichment of proteins in USC-Exos, such as DMBT1 and TIMP1 (13) and CTHRC1 and OPG (14), is involved in the regulation of bone metabolism during the therapeutic process. These verified molecular mechanisms are also applicable to our current research because our research also investigated bone metabolism. However, we should refer to the current research results and explore and verify new functional proteins or miRNAs in USC-Exos in our follow-up experiments to provide a comprehensive and in-depth study about the mechanism of osteolysis. We also noticed that comparing with sited reference, there is a different result in western blot analysis of exosomal markers in USCs and USC-Exos presented by previously published article (14). However, our results are consistent with those of other researchers (38–41). USCs produce and secrete exosomes to the extracellular environment, and the method to collect and isolate USC-Exos from the culture medium is the exosomal enrichment process, hence the different protein quantities and contents in USCs and USC-Exos may be the main reason for the difference in western blot analysis of exosomal markers in USCs and USC-Exos. Besides the individual difference between the donors, the different passage of USCs, the different loading amount of the protein, or the different voltage value and current value, et al., to perform the western blot analysis also may have influence on the different results.

In summary, USC-Exos incorporated into PLGA microspheres showed great potential in ameliorating particulate polyethylene-induced osteolysis. According to a previous study, exosomes can be easily endocytosed into recipient cells because of their cup-shaped structure with a lipid bilayer, nanosize and favorable density (42). Thus, exosomes have shown great advantages in the field of nanomedicine (43). Researchers and clinicians have developed multiple clinical trials investigating the therapeutic effects of exosomes; however, no effective and safe drug has been approved, which may be due to the insufficient knowledge of exosomes and their bioactive components (44). Additionally, regarding the clinical application of USC-Exos in preventing and treating periprosthetic osteolysis, a standard process for the isolation, purification and storage of exosomes with low cost and degradation should be established (45). Furthermore, synthesizing new multifunctional bioactive materials with long-term and sustained exosome release and maintaining exosome integrity and bioactivity throughout the process of encapsulation and release are vital to promote tissue repair and regeneration (46, 47).

## Conclusion

We successfully fabricated NPs with long-term and sustained exosome release for orthopedic wear debris particle-induced osteolysis treatment. Our study presents a new strategy to synthesize PLGA-loaded exosome bioactive NPs for the delivery of therapeutic agents.

## Data Availability Statement

All the data supporting the findings of this study are available within the paper and its supplementary information. Raw data for the figures in this study are available from the authors upon request.

## Ethics Statement

This study was approved by the Department of Laboratory Animal Management Committee of Central South University (No. 2019030499) and was conducted according to all current ethics guidelines.

## Author Contributions

JX and YH conceived and designed the experiments. HL, MZ, JY, XF, and YW performed the experiments. HL analyzed the data and prepared all the figures. HL, YH, and JX drafted the manuscript. All authors reviewed and approved the manuscript.

## Funding

This work was supported by the National Natural Science Foundation of China (Grant No. 81974339) and the Science and Technology Plan Project of Hunan Province (Grant No. 2019JJ40499).

## Conflict of Interest

The authors declare that the research was conducted in the absence of any commercial or financial relationships that could be construed as a potential conflict of interest.

## Publisher's Note

All claims expressed in this article are solely those of the authors and do not necessarily represent those of their affiliated organizations, or those of the publisher, the editors and the reviewers. Any product that may be evaluated in this article, or claim that may be made by its manufacturer, is not guaranteed or endorsed by the publisher.
