# Supplementary figures and images for: Exosomes From Human Urine-Derived Stem Cells Encapsulated Into PLGA Nanoparticles for Therapy in Mice With Particulate Polyethylene-Induced Osteolysis
Source: Front Med (Lausanne). 2021 Dec 6;8:781449. doi: 10.3389/fmed.2021.781449 (PMC8685253; doi:10.3389/fmed.2021.781449)

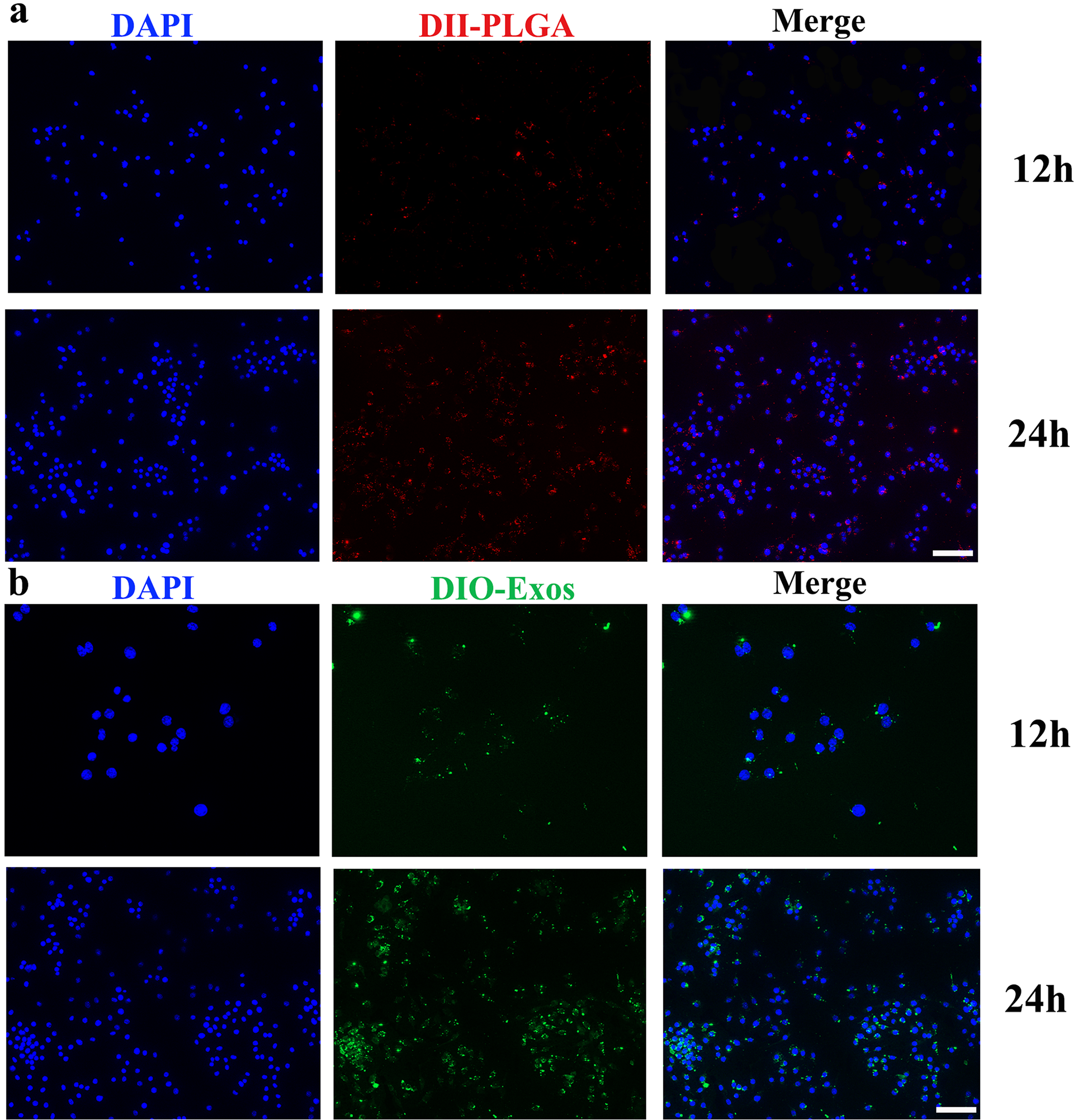

Supplement: Supplementary file 1 [file Image_1.TIF]

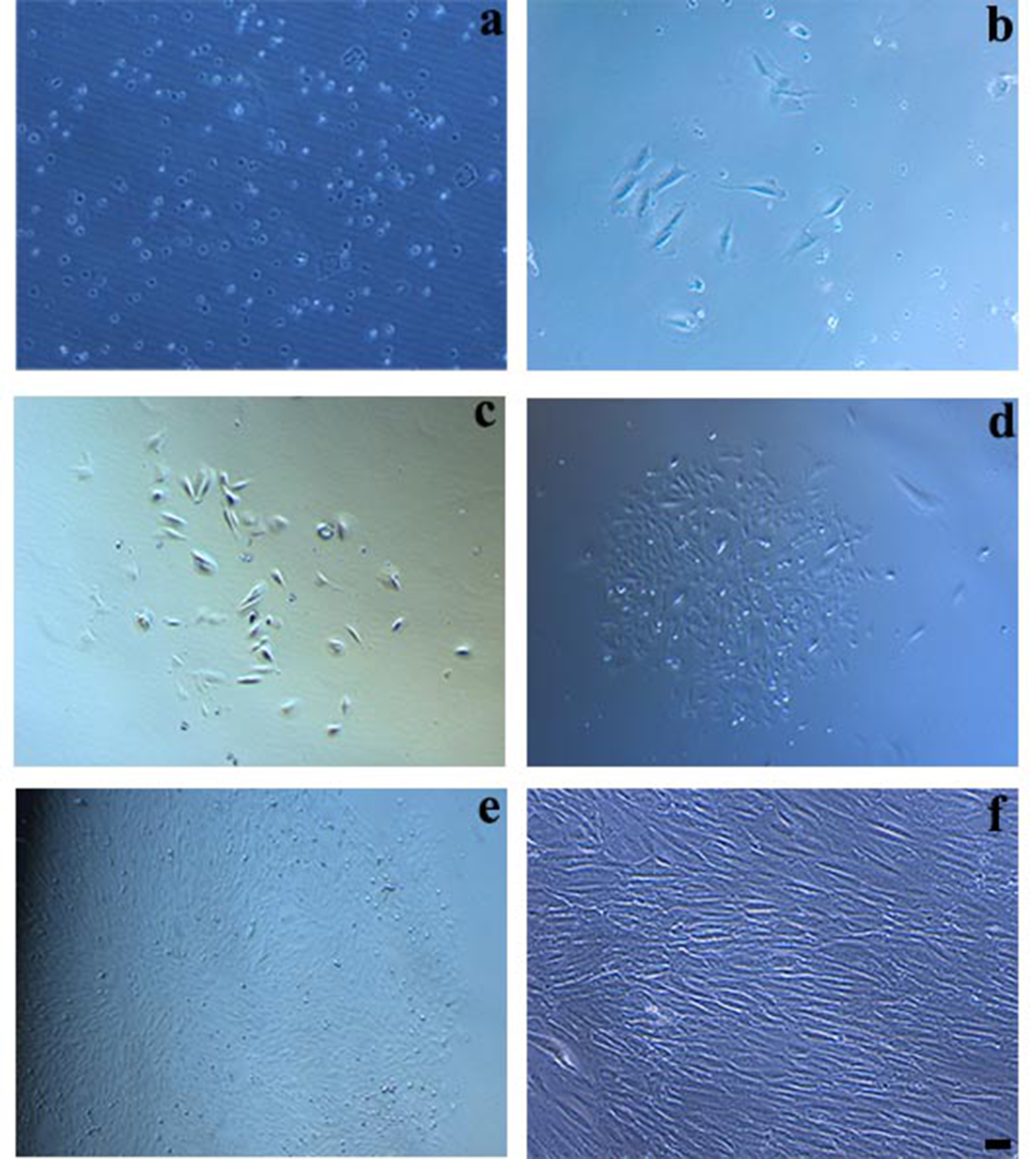

Supplement: Supplementary file 2 [file Image_2.TIF]
